# Assessment of the suitability of biodegradable rods for use in posterior lumbar fusion: An in-vitro biomechanical evaluation and finite element analysis

**DOI:** 10.1371/journal.pone.0188034

**Published:** 2017-11-16

**Authors:** Fon-Yih Tsuang, Yueh-Ying Hsieh, Yi-Jie Kuo, Chia-Hsien Chen, Feng-Huei Lin, Chen-Sheng Chen, Chang-Jung Chiang

**Affiliations:** 1 Institute of Biomedical Engineering, National Taiwan University, Taipei City, Taiwan; 2 Division of Neurosurgery, Department of Surgery, National Taiwan University Hospital, Taipei City, Taiwan; 3 Department of Traumatology, National Taiwan University Hospital, Taipei City, Taiwan; 4 Department of Orthopedics, Shuang Ho Hospital, Taipei Medical University, New Taipei City, Taiwan; 5 Department of Orthopedics, Taipei Medical University Hospital, Taipei City, Taiwan; 6 Department of Orthopedics, School of Medicine, College of Medicine, Taipei Medical University, Taipei City, Taiwan; 7 Division of Medical Engineering, National Health Research Institute, Miaoli County, Taiwan; 8 Department of Physical Therapy and Assistive Technology, National Yang-Ming University, Taipei, Taiwan; 9 College of Biomedical Engineering, Taipei Medical University, Taipei, Taiwan; Kanazawa University, JAPAN

## Abstract

Interbody fusion with posterior instrumentation is a common method for treating lumbar degenerative disc diseases. However, the high rigidity of the fusion construct may produce abnormal stresses at the adjacent segment and lead to adjacent segment degeneration (ASD). As such, biodegradable implants are becoming more popular for use in orthopaedic surgery. These implants offer sufficient stability for fusion but at a reduced stiffness. Tailored to degrade over a specific timeframe, biodegradable implants could potentially mitigate the drawbacks of conventional stiff constructs and reduce the loading on adjacent segments. Six finite element models were developed in this study to simulate a spine with and without fixators. The spinal fixators used both titanium rods and biodegradable rods. The models were subjected to axial loading and pure moments. The range of motion (ROM), disc stresses, and contact forces of facet joints at adjacent segments were recorded. A 3-point bending test was performed on the biodegradable rods and a dynamic bending test was performed on the spinal fixators according to ASTM F1717-11a. The finite element simulation showed that lumbar spinal fusion using biodegradable implants had a similar ROM at the fusion level as at adjacent levels. As the rods degraded over time, this produced a decrease in the contact force at adjacent facet joints, less stress in the adjacent disc and greater loading on the anterior bone graft region. The mechanical tests showed the initial average fatigue strength of the biodegradable rods was 145 N, but this decreased to 115N and 55N after 6 months and 12 months of soaking in solution. Also, both the spinal fixator with biodegradable rods and with titanium rods was strong enough to withstand 5,000,000 dynamic compression cycles under a 145 N axial load. The results of this study demonstrated that biodegradable rods may present more favourable clinical outcomes for lumbar fusion. These polymer rods could not only provide sufficient initial stability, but the loss in rigidity of the fixation construct over time gradually transfers loading to adjacent segments.

## Introduction

Spinal fusion is a common and effective method for treating degenerative lumbar disc disease. In addition, instrumentation with a spinal fixator can provide immediate spinal stability and facilitate the fusion process[1, 2]. However, a number of complications have been reported in literature after spinal fusion; adjacent segment degeneration (ASD), hardware-related pain and infections, to name a few. Radcliff et al. [3] reported a 30% incidence of ASD after anterior lumbar interbody fusion (ALIF) and posterior instrumentation. In a study by Kumar et al. [4], 31 out of 83 patients (36.1%) presented radiographic ASD above the fusion level, and 16.8% of patients needed a secondary surgery. Etebar et al. [5] reviewed the results of 125 patients who underwent lumbar fusion with instrumentation and 15% of these patients were found to have developed symptomatic ASD. Some studies [6,7] have suggested that rigid fixation leads to increased stresses on the intervertebral discs and facet joints at adjacent levels, and the increased loading over time could cause regional hypermobility, facet hypertrophy, and disc degeneration at adjacent segments. It is also believed that the increased loading on the facet joints at adjacent levels might be a key factor in the development of ASD [8,9,10].

Persistent lower back pain is commonly experienced by patients after spinal fusion. Approximately 10–15% of patients require a secondary operation due to hardware-related back pain [11,12]. In addition, corrosion debris and the resulting inflammatory responses around the hardware have been implicated as potential sources of pain [13,14]. The fretting corrosion between screws and rods can release ions and activate the immune system by forming metal-protein complexes, which could subsequently lead to metal hypersensitivity responses. To tackle this, Zotti et al. [15] found that removing pedicle screws in patients with persistent lower back pain after solid fusion produced good to excellent clinical outcomes. Jeon et al. [16] analyzed clinical and radiological results after solid fusion of thoracolumbar fractures, and the results showed that removing internal fixators could significantly alleviate patient pain and disability, but the precise mechanism behind this was not clear. A lumbosacral finite element model developed by Hsieh et al. [17] showed that removal of spinal fixators after fusion could decrease the negative impact on adjacent segments. Their result suggested that a higher Young's modulus of the instrumented metal and an increased stiffness of the fusion site are potential sources of stress concentrations at the adjacent levels, especially at the cephalic level. Although removing pedicle screws has been shown to alleviate some of the complications with spinal fusion, the patient must undergo a secondary operation for this. As with any operation, there is an element of risk involved and associated morbidity, including physiological stress under anesthesia, residual wound problems, neurovascular injury, and surgical site infection.

Biodegradable implants break down gradually over time when placed in the body. As the implant degrades, loading is transferred to the surrounding anatomical structures, and so could be expected to have lower levels of stress shielding [18–20]. Therefore, biodegradable rods may be an attractive alternative for use in posterior lumbar fixation because of the ability to gradually transfer loads from the fixator to the fusion mass during the fusion process. Bezer et al. [18] used poly-lactides (PLLA) to construct spinal rods for posterolateral fusion in rabbits and demonstrated that both PLLA rods and K-wires provided enough stability to facilitate spinal fusion. Similarly, Johnsson et al. [19] accomplished successful posterolateral spinal fusion using biodegradable rods.

The aim of this study is to assess the biomechanical properties of biodegradable rods at different time points when used as posterior lumbar fixators. The results will be compared to conventional titanium spinal fixators. Finite element analysis (FEA) and in-vitro biomechanical evaluation will be used to investigate whether biodegradable rods present a reliable option for spinal fusion, and if the complications at adjacent segments could be mitigated by using such biodegradable implants.

## Materials and methods

### FE models of the lumbar spine and implants

**Fig 1:**
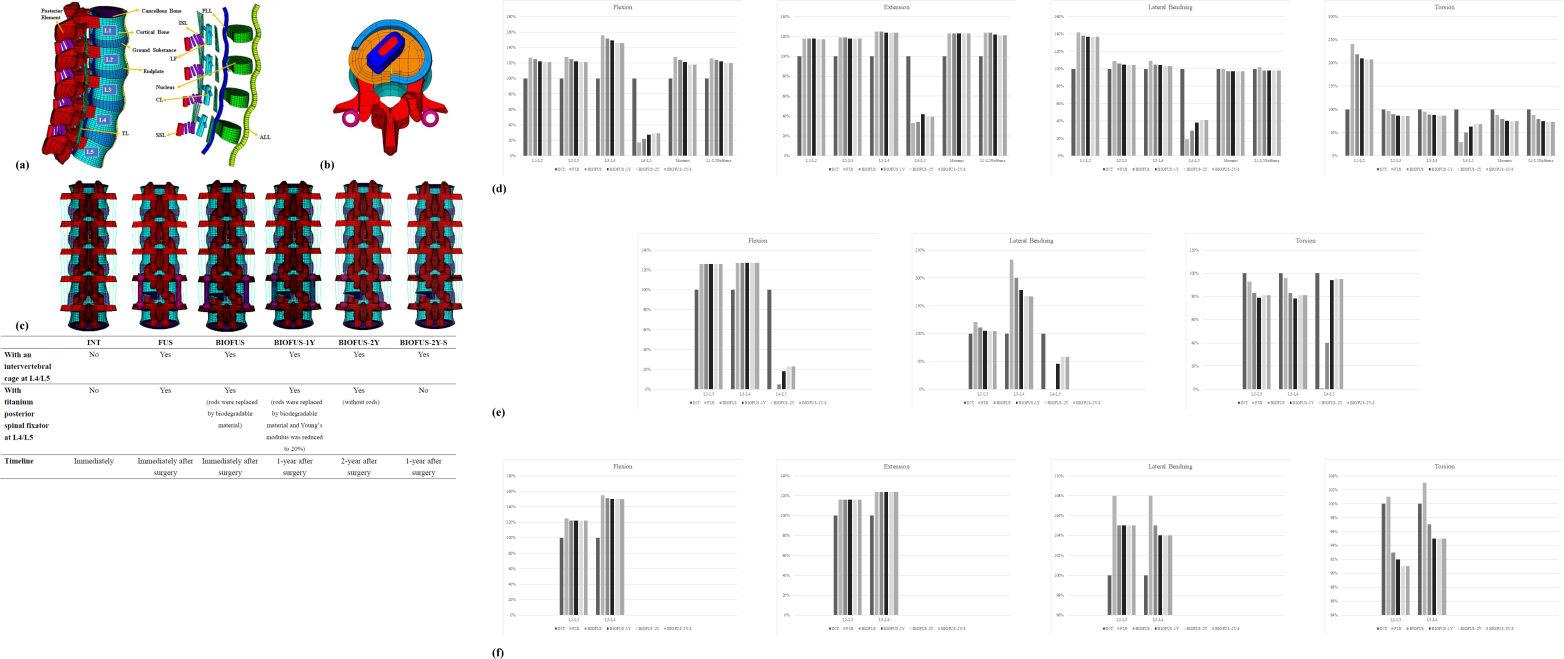
FE models of the spine with and without implants. a) The osseous structures, intervertebral discs, and ligaments of the intact spine. b) At the L4-L5 disc space, the cage was placed obliquely with the left posterolateral corner of the annulus fibrosus removed, as in PLIF procedures. c) Six FE models used in this study. The d) ROM, e) facet joint forces, f) disc stresses of all models normalized by the INT model.

The finite element software ANSYS (ANSYS Inc., Canonsburg, PA, USA) was used to create an FE model of a 5-level intact lumbar spine. As shown in Fig 1A, a normal human lumbar spine consists of osseoligamentous L1–L5 vertebrae, intervertebral discs, endplates, posterior bony elements, and 7 ligaments. The intervertebral disc (IVD) consists of an annulus fibrosus and nucleus pulposus, with 12 double-cross-linked fibrous layers embedded in a ground substance. The annulus material was modeled based on an incompressible, hyperelastic, 2-parameter (C1, C2) Mooney-Rivlin formulation, and the nucleus pulposus was modeled as an incompressible fluid. Convergence testing and model validation was performed in previous studies [21–22], and the results were shown to correspond to other comparable published FE models [23]. A more detailed description of the spinal model used in this study and its material properties was presented in an earlier publication [21].

FE models of the biodegradable spinal rods (self-reinforced copolymer rod, Inion Oy, Tampere, Finland) [20] and titanium spinal rods were incorporated into the CB PROT II Posterior Spinal System (Chin Bone Corp., Taiwan; US FDA 510(k): K142655), which consists of titanium alloy screws (diameter 5.5 mm) connected by vertical rods. The lumbar intervertebral cage was modeled from the ReBorn Essence cage (Baui Biotech Co. Ltd., New Taipei City, Taiwan) made of PEEK.

All implant components (pedicle screws, titanium spinal rods, biodegradable spinal rods, and lumbar intervertebral cage) were modeled using 8-node solid elements. The lumbar intervertebral cage was implanted using a posterior lateral oblique approach. As shown in Fig 1B, the left posterolateral corner of the L4-L5 annulus fibrosus was also removed so as to simulate the normal procedure for posterior lateral interbody fusion [24].

Six finite element models were developed in this study:

1. Intact lumbar spine (INT) without any implants.
2. Lumbar spine implanted with an intervertebral cage and conventional posterior spinal fixator with titanium rods at L4-L5 to simulate a fusion segment (FUS). Timeline is immediately after surgery.
3. Lumbar spine implanted with an intervertebral cage and posterior spinal fixator with biodegradable rods at L4-L5 (BIOFUS) (Fig 1C). Timeline is immediately after surgery.
4. BIOFUS model with the Young’s modulus of the biodegradable rods reduced to 20% (determined from 3-point bending testing) to simulate degradation after being implanted for 1 year (BIOFUS-1Y).
5. BIOFUS model without biodegradable rods (BIOFUS-2Y) that has achieved complete fusion after 2 years after surgery. The model still retains the pedicle screws.
6. BIOFUS model without biodegradable rods or pedicle screws (BIOFUS-2Y-S) that has achieved complete fusion 2 years after surgery.

The interfaces between facet articular surfaces were treated as standard contact pairs at all levels. The spinal fusion segment was defined as two adjacent vertebrae bridged with pedicle screws and rods in combination with the intervertebral cage. All FE models were fixed at the base of the fifth vertebrae. A hybrid method demonstrated by Panjabi was used to evaluate the effects on the adjacent spinal levels [25]. Loads were applied to the models in two steps. In the first loading step, an axial load of 150 N was applied perpendicular to the upper surface of the first lumbar vertebra. In the second loading step, a pure unconstrained moment was applied to the upper surface of the first lumbar vertebra in 0.36 Nm increments to ensure the resultant ROMs (L1-L5) of all FE models could achieve 16 degrees in flexion, 9 degrees in extension, 17 degrees in left torsion, and 22 degrees in left lateral bending. Table 1 details the resultant ROMs of the instrumented level and adjacent level and the segment stiffness of L1-L5 for each model.

**Table 1:** ROM of six FE models at all motion segments.

Motion	Model	L1-L2 (Degree)	L2-L3 (Degree)	L3-L4 (Degree)	L4-L5 (Degree)	Moment (Nm)	L1-L5 Stiffness (Nm/Degree)
Flexion	INT	4.45 (100%)	4.43 (100%)	4.34 (100%)	5.78 (100%)	8.7 (100%)	0.46 (100%)
	FUS	5.66 (127%)	5.65 (128%)	6.78 (156%)	1.01 (17%)	11.1 (128%)	0.58 (126%)
	BIOFUS	5.56 (125%)	5.54 (125%)	6.61 (152%)	1.26 (22%)	10.8 (124%)	0.57 (124%)
	BIOFUS-1Y	5.44 (122%)	5.41 (122%)	6.45 (149%)	1.57 (27%)	10.5 (121%)	0.56 (122%)
	BIOFUS-2Y	5.40 (121%)	5.38 (121%)	6.32 (146%)	1.65 (29%)	10.3 (118%)	0.55 (120%)
	BIOFUS-2Y-S	5.40 (121%)	5.38 (121%)	6.32 (146%)	1.65 (29%)	10.3 (118%)	0.55 (120%)
Extension	INT	3.05 (100%)	2.62 (100%)	2.56 (100%)	2.57 (100%)	7.80 (100%)	0.72 (100%)
	FUS	3.60 (118%)	3.11 (119%)	3.19 (125%)	0.84 (33%)	9.60 (123%)	0.89 (124%)
	BIOFUS	3.59 (118%)	3.11 (119%)	3.20 (125%)	0.87 (34%)	9.60 (123%)	0.89 (124%)
	BIOFUS-1Y	3.59 (118%)	3.10 (118%)	3.18 (124%)	1.07 (42%)	9.60 (123%)	0.88 (122%)
	BIOFUS-2Y	3.58 (117%)	3.09 (118%)	3.17 (124%)	1.01 (39%)	9.60 (123%)	0.87 (121%)
	BIOFUS-2Y-S	3.58 (117%)	3.09 (118%)	3.17 (124%)	1.01 (39%)	9.60 (123%)	0.87 (121%)
Lateral Bending	INT	5.74 (100%)	5.01 (100%)	4.70 (100%)	4.48 (100%)	9.90 (100%)	0.50 (100%)
	FUS	8.14 (142%)	5.48 (109%)	5.11 (109%)	0.85 (19%)	9.90 (100%)	0.51 (102%)
	BIOFUS	7.91 (138%)	5.32 (106%)	4.95 (105%)	1.28 (29%)	9.6 (97%)	0.49 (98%)
	BIOFUS-1Y	7.88 (137%)	5.28 (105%)	4.90 (104%)	1.7 (38%)	9.6 (97%)	0.49 (98%)
	BIOFUS-2Y	7.86 (137%)	5.23 (104%)	4.85 (103%)	1.82 (41%)	9.58 (97%)	0.49 (98%)
	BIOFUS-2Y-S	7.86 (137%)	5.23 (104%)	4.85 (103%)	1.82 (41%)	9.58 (97%)	0.49 (98%)
Torsion	INT	2.01 (100%)	2.30 (100%)	2.68 (100%)	3.75 (100%)	9.90 (100%)	0.92 (100%)
	FUS	4.84 (241%)	2.23 (97%)	2.54 (95%)	1.14 (30%)	8.70 (88%)	0.81 (88%)
	BIOFUS	4.38 (218%)	2.07 (90%)	2.39 (89%)	1.86 (50%)	7.80 (79%)	0.73 (79%)
	BIOFUS-1Y	4.22 (210%)	2.00 (87%)	2.35 (88%)	2.31 (62%)	7.50 (76%)	0.69 (75%)
	BIOFUS-2Y	4.18 (208%)	1.96 (85%)	2.33 (87%)	2.55 (68%)	7.42 (75%)	0.67 (73%)
	BIOFUS-2Y-S	4.18 (208%)	1.96 (85%)	2.33 (87%)	2.55 (68%)	7.42 (75%)	0.67 (73%)

The percentages indicate the ROM of all models normalized by the ROM of INT

**Fig 2:**
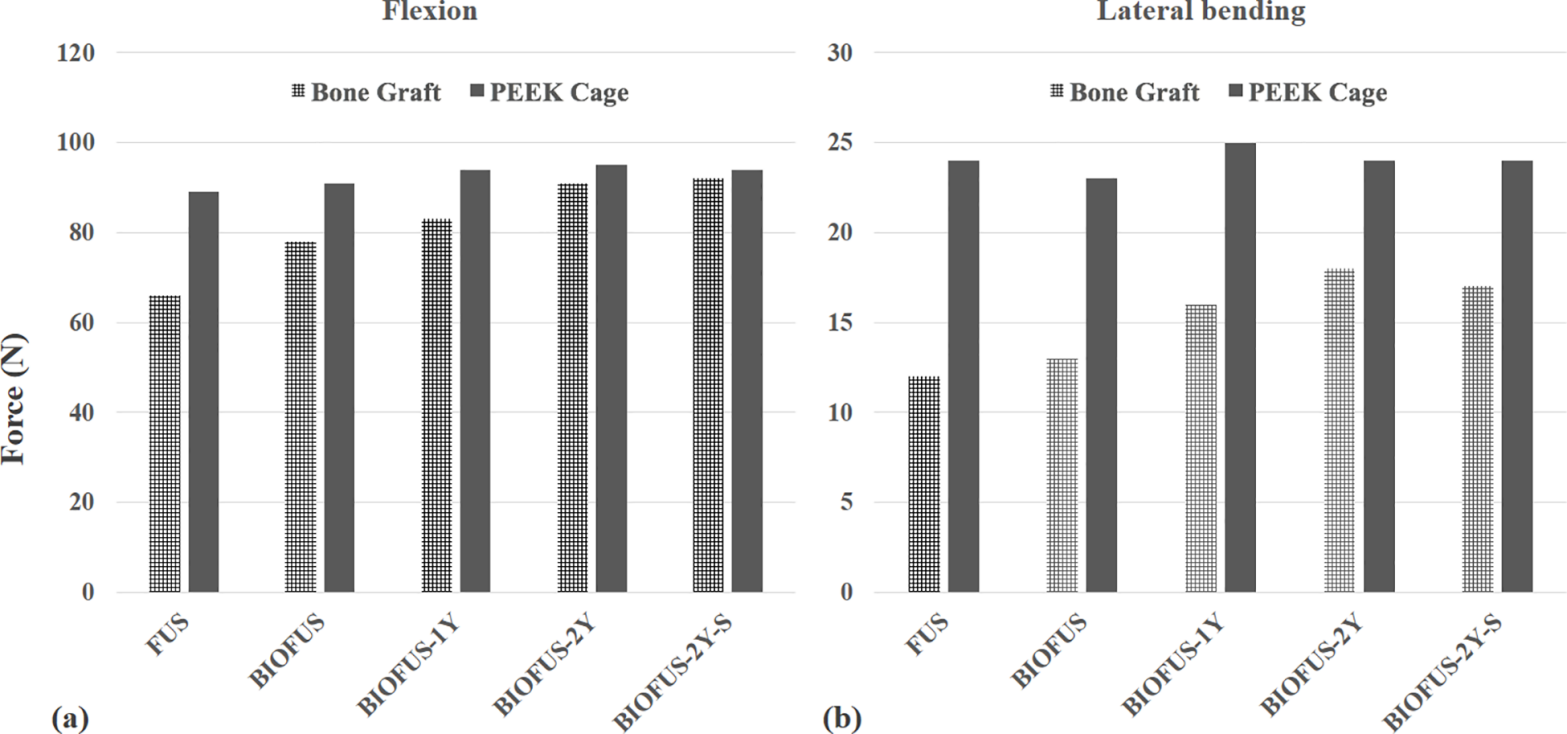
Loading on cage and bone grafts in each group. a) in flexion and b) in lateral bending.

This study analyzed the ROM of each motion segment, the facet contact forces (FCFs) and the peak disc stresses at L2-3/L3-4 under flexion, extension, torsion, and left lateral bending. The results are presented in Tables 1, 2 and 3. The loading on the cage and bone graft in each model during flexion and lateral bending is shown in Fig 2.

**Table 2:** Facet joint forces in instrumented levels and cephalic adjacent levels.

Motion	Model	L2-L3 (N)	L3-L4 (N)	L4-L5
Extension	INT	65 (100%)	71 (100%)	66 (100%)
	FUS	82 (126%)	90 (127%)	0 (0%)
	BIOFUS	82 (126%)	90 (127%)	3 (5%)
	BIOFUS-1Y	82 (126%)	90 (127%)	12 (18%)
	BIOFUS-2Y	82 (126%)	90 (127%)	15 (23%)
	BIOFUS-2Y-S	82 (126%)	90 (127%)	15 (23%)
Lateral Bending	INT	19 (100%)	9 (100%)	13 (100%)
	FUS	23 (121%)	21 (233%)	0 (0%)
	BIOFUS	21 (111%)	18 (200%)	0 (0%)
	BIOFUS-1Y	20 (105%)	16 (178%)	6 (46%)
	BIOFUS-2Y	19.8 (104%)	15 (167%)	7.5 (58%)
	BIOFUS-2Y-S	19.8 (104%)	15 (167%)	7.5 (58%)
Torsion	INT	125 (100%)	124 (100%)	112 (100%)
	FUS	116 (93%)	119 (96%)	1 (1%)
	BIOFUS	104 (83%)	103 (83%)	45 (40%)
	BIOFUS-1Y	99 (79%)	97 (78%)	105 (94%)
	BIOFUS-2Y	101 (81%)	100 (81%)	106 (95%)
	BIOFUS-2Y-S	101 (81%)	100 (81%)	106 (95%)

The percentages indicate the facet joint forces of all models normalized by the facet joint forces of INT

**Table 3:** Disc stresses at cephalic adjacent levels.

Motion	Model	L2-L3 (KPa)	L3-L4 (KPa)
Flexion	INT	880 (100%)	742 (100%)
	FUS	1,100 (125%)	1,150 (155%)
	BIOFUS	1,070 (122%)	1,120 (151%)
	BIOFUS-1Y	1,070 (122%)	1,110 (150%)
	BIOFUS-2Y	1,070 (122%)	1,110 (150%)
	BIOFUS-2Y-S	1,070 (122%)	1,110 (150%)
Extension	INT	398 (100%)	424 (100%)
	FUS	460 (116%)	525 (124%)
	BIOFUS	460 (116%)	526 (124%)
	BIOFUS-1Y	460 (116%)	524 (124%)
	BIOFUS-2Y	460 (116%)	524 (124%)
	BIOFUS-2Y-S	460 (116%)	524 (124%)
Lateral Bending	INT	951 (100%)	906 (100%)
	FUS	1,030 (108%)	975 (108%)
	BIOFUS	1,000 (105%)	950 (105%)
	BIOFUS-1Y	996 (105%)	943 (104%)
	BIOFUS-2Y	995 (105%)	941 (104%)
	BIOFUS-2Y-S	995 (105%)	941 (104%)
Torsion	INT	314 (100%)	345 (100%)
	FUS	316 (101%)	355 (103%)
	BIOFUS	293 (93%)	335 (97%)
	BIOFUS-1Y	288 (92%)	329 (95%)
	BIOFUS-2Y	286 (91%)	327 (95%)
	BIOFUS-2Y-S	286 (91%)	327 (95%)

The percentages indicate the disc stresses of all models normalized by the disc stresses of INT

### Mechanical testing

**Fig 3:**
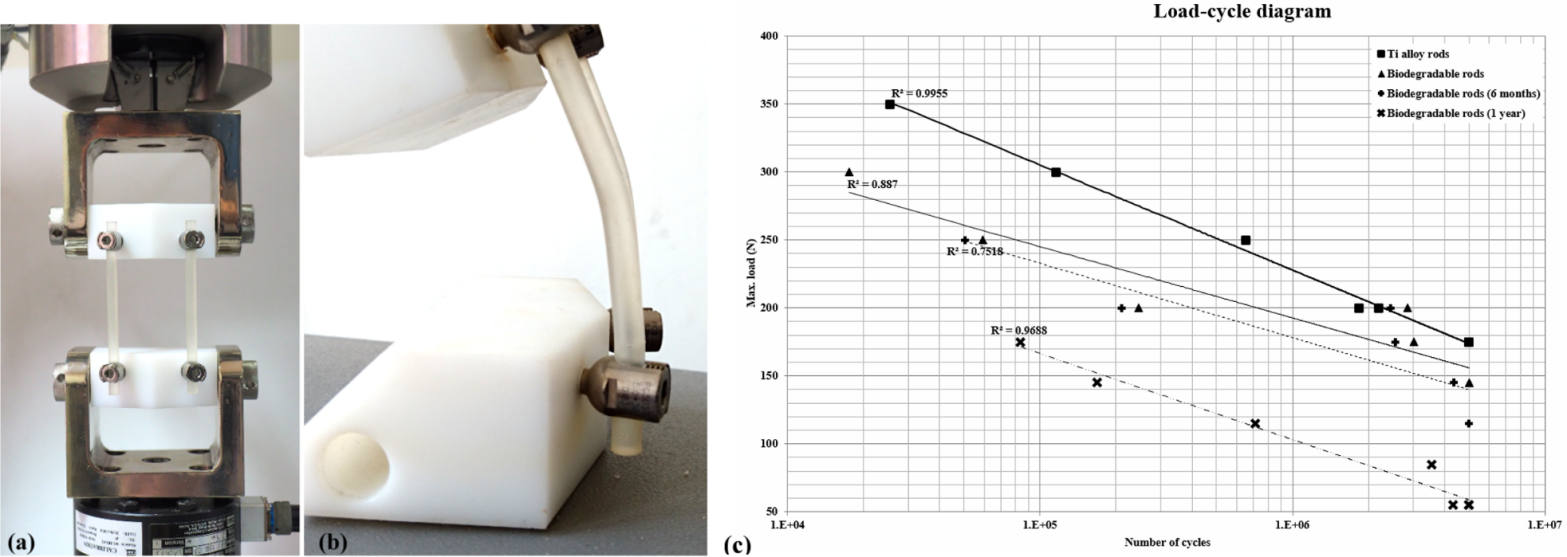
Safety testing according to ASTM Standard F1717-96. a) photo of testing sample, b) the failure mode for biodegradable rods (plastic deformation), c) axial compression bending fatigue curves for CB PROT II with metal rods, biodegradable rods, and rods degraded for 1 year.

Blocks of ultra-high molecular weight polyethylene (UHMWPE) were formed according to ASTM F1717-11a. As shown in Fig 3A, two screws were secured in each block and linked by biodegradable rods or titanium rods. This setup simulated fixation between two adjacent vertebrae. The constructs (rods, screws and cages) were setup according to the manufacturer’s instructions using standard surgical instruments. The nuts were tightened at a torque of 12 Nm for the titanium rods, and at 7 Nm for the biodegradable rods. The active length was 76 mm, and the moment arm from the centerline to insertion point was 40mm in the x-axis and 20 mm in y-axis. The test construct was fixed in a material testing system (MTS 370 series) frame with an axial/torsional load cell (model 662.20H-05). The axial and torsional capabilities were 25 KN and 250 Nm, respectively.

The dynamic compression bending tests were performed according to the methods described in ASTM F1717-11a, and 7 samples were tested for each construct [26]. All tests were done in dry air at room temperature. For the dynamic fatigue test, the frequency was set at 5 Hz with a cyclic sine wave. The R value (minimum load divided by maximum load) was 10. The end point of the test was when the sample failed (meaning any permanent deformation that altered the functional performances), the distance between the test blocks was reduced to less than 38 mm, or 5,000,000 cycles was reached. The loads applied and the durations of loading were recorded to determine the fatigue strength.

A total of 14 biodegradable rods and 14 titanium rods were used in this test; 2 rods in each construct. The biodegradable rods were made of medical grade biodegradable materials composed of rigid and elastic copolymer components. The titanium rods are commercially available in the CB PROT II Posterior Spinal System. The dimensions of these two rods are identical; diameter 5.5 mm, length 105 mm. The pedicle screws (CB PROT II Posterior Spinal System) used in this study were a medical grade titanium alloy (Ti6Al4V) with a diameter of 5.0 mm and length of 30 mm.

3-point bending testing and fatigue testing was performed on six biodegradable rods and 14 fusion constructs (rods, screws, and test blocks) according to ISO 178:2001 and ASTM D790-10. To simulate polymer degradation in the human body, the test samples were soaked in phosphate buffered saline (PBS) at a temperature of 37°C and pH of 7.4. Testing was done at three specific points in time: immediately after the constructs were set up (i.e. immediately after surgery), after soaking for 6 months and after soaking for 12 months.

## Results

### FE models of the lumbar spine and implants

#### ROM of each level

Table 1 shows that the ROM decreased at the fusion site and increased at adjacent segments in all implanted models. In comparison to the intact (INT) model, the ROM of the FUS models was greater than the BIOFUS models. As time went on, the ROM at adjacent segments in the biodegradable rod groups (BIOFUS-1Y, BIOFUS-2Y and BIOFUS-2Y-S) was relatively similar to the intact (INT) spine under all loading conditions.

#### Contact force at adjacent facet joint

The increased contact force ratio on the adjacent facet joint at L2/L3 and L3/L4 under the different loading conditions is shown in Table 2. In all fusion models, the ratio at the L3/L4 facet in extension was greater than at the L2/L3 facet. The contact force at adjacent facet joints in all biodegradable rod models was lower than in the titanium rod models. However, the BIOFUS-2Y-S and BIOFUS-2Y models did demonstrate a similar contact force at adjacent facet joints. Generally, the contact force at adjacent facet joints decreased as the rods degraded over time.

#### Peak stress at adjacent disc

Table 3 details the increase in the ratio of peak stress on the intervertebral discs (IVDs) at L2/L3 and L3/L4 under different loading conditions. In all fusion models, the peak stresses on the IVDs at adjacent levels were considerably higher than in the intact model. In addition, the stress ratio at the L3/L4 disc was greater than at the L2/L3 disc. The BIOFUS-2Y and BIOFUS-2Y-S models had the lowest ratio of stress increase of all models. The FUS model showed the greatest change in stress at the IVD (at both L2/L3 or L3/L4), with the intervertebral stress at L3/L4 increasing by 55% in flexion. All biodegradable rod models had lower increased ratios of peak stress on the IVDs at the adjacent levels than the titanium rod (FUS) models. The increased ratios got lower while the elastic modulus of the rods decreased, which represented the degradation of the rods.

#### Comparison of loading on bone grafts and cages

Fig 2 demonstrates the forces on the bone grafts and PEEK cages under various loading conditions when using biodegradable rods and titanium rods. Immediately after surgery, the loading on the bone grafts in the BIOFUS models exceeded the FUS models by 18% in flexion and 8% in lateral bending. At 1 year after surgery, the loading on the bone grafts in BIOFUS-1Y exceeded the FUS models by 26% in flexion and 33% in lateral bending. Under all motion conditions, the loading on the bone grafts in BIOFUS-2Y were almost equivalent to BIOFUS-2Y-S.

### Mechanical testing for safety evaluation

The 3-point bending test showed the Young’s modulus of the biodegradable rods decreased by 20% and 80% at 6 months and 12 months after surgery.

The results of the dynamic compression bending tests are presented in Fig 3. The CB PROT II Posterior Spinal System with titanium rods had a fatigue strength of 175 N. In contrast, the fatigue strength of the biodegradable rods was only 145 N, and this decreased to 115 N and 55 N after soaking in solution for 6 and 12 months. The constructs with titanium rods primarily failed at the pedicle screws, whereas the constructs with biodegradable rods primarily failed by plastic deformation of the rods (Fig 3B). The R^2^ values of the titanium alloy rods, biodegradable rods, biodegradable rods soaked for 6 months, and biodegradable rods soaked for 1 year were 0.9955, 0.887, 0.7518, and 0.9688 respectively (Fig 3C).

## Discussion

This study demonstrated how biodegradable implants may offer superior outcomes for lumbar spine fusion over conventional metal spinal implants, including a lower contact force at adjacent facet joints, lower peak stresses in the adjacent disc and greater loading on the anterior bone graft region. Savage et al. [20] found that biodegradable rods produced less stress shielding and offered better dynamic loading in the spine over titanium rods. In addition to mechanical advantages, the radiolucent feature of resorbable rods could allow for better imaging and potentially eliminate surgical complications associated with removing metallic implants.

To the best of our knowledge, this is the first publication to evaluate the effectiveness of biodegradable rods in posterior lumbar fixation. This study assessed the biomechanical behavior and mechanical properties of 5.5 mm diameter copolymer rods using finite element analysis and mechanical tests. An FE model was used to demonstrate the changes of ROM at adjacent levels and the changes of the stress/force distribution on IVDs and facet joints at adjacent levels. Mechanical tests were used to evaluate the properties of the rods themselves.

The FE analysis showed no significant differences in ROM and stress on adjacent facets and discs between the titanium rod model and biodegradable rod models. We believe that the PEEK interbody cage used in this study is an important factor in determining the increase in stress and ROM at adjacent levels. Because of the greater Young’s modulus of the PEEK cage, replacing an intervertebral disc with a cage would increase the stiffness of the fusion segment, which could induce greater motion and stress on the adjacent discs [17]. Although there was no significant difference in ROM and stress at the adjacent discs between the models implanted with titanium rods and biodegradable rods, the BIOFUS-2Y-S and BIOFUS-2Y models still had less of an impact on adjacent levels, especially in extension and rotation.

Studies [27–29] have shown that greater stiffness at the fusion levels increases the stress on adjacent facet joints. These increased loads could lead to segmental hypermobility, facet hypertrophy, and IVD degeneration at the adjacent segments. Faizan et al. reported the significant increase in stress after fusion by using finite element analysis [28]. In this current study, the contact force at the adjacent facet joints during flexion was zero in each group because the facets moved away from each other as the spine flexed. For the FUS model, the facet contact forces at L2/L3 and L3/L4 increased 1.21 and 2.33 times over those of the intact model when placed in a bending motion. After the biodegradable rods degraded, there was a notable decrease in the stiffness of fusion segment The contact forces at the adjacent facet joints also decreased compared to the FUS model. On the other hand, both the BIOFUS-2Y-S and BIOFUS-2Y models demonstrated a reduction in contact forces at the adjacent facet joints, especially in extension. Greater loading at the adjacent facets may induce joint degeneration, which has been suggested to be an initiating factor for adjacent segment disease [30]. Reducing the stiffness of the fusion segment could decrease the loading on the adjacent facets, which may in turn slow down the degeneration of the adjacent segment. The FE models in this study showed a significant decrease in loading at the adjacent facet joints both after removing the pedicle screws and after complete resorption of the biodegradable rods. These two situations may potentially reduce the incidence of adjacent segment disease.

The FE analysis also indicated that the loading on the bone grafts in the titanium rod models was less than in the biodegradable rod models. Due to lower stiffness and the resorptive properties of the biodegradable rods, there would be less of a stress-shielding effect in the posterior construct. As the rods degrade, compression loading on the anterior bone graft may promote bone integration between the endplates and cancellous bone over time. Research [31, 32] has shown that lower stress-shielding can enhance the process of interbody fusion. As the load is gradually transferred from the posterior fixator to the anterior vertebral body the stress on adjacent facet joints is reduced, which could potentially slow down the degeneration of the adjacent level.

The mechanical test showed that the biodegradable rods could withstand 5,000,000 dynamic compression cycles under a 145 N axial load, which is less than the fatigue strength (175 N axial load) of the titanium rods. After 6 months and 12 months of soaking in solution, the fatigue strength of the biodegradable rods gradually decreased to 115 N and 55 N, respectively. However, the rods retained enough strength and rigidity to stabilize the spine during the bone fusion process.

There are some limitations to this study that should be noted. First, only single-level fusion of the lumbar spine was simulated. Second, the structure of the vertebral bodies was assumed as isotropic and homogenous, which does not fully reflect the true properties of bone. Third, the loading conditions in this study were not truly physiological because the mechanical effects of muscle and soft tissue contractions were not considered. The spine is an exquisitely complex structure, so these FE models were adapted to simplify the geometry and conditions. However, while not entirely representative of physiological conditions, such computational methods may still provide accurate and reliable results that are not subject to individual variations in anatomy. Therefore, this study provides useful data around mechanical differences between biodegradable rods and titanium rods when used as internal fixators for interbody fusion surgery.

## Conclusion

Spinal fixators impose greater constraint on the bridged segment than fusion alone. This current study demonstrated how biodegradable rods may be used as an ideal spinal fixator. They provide immediate stability after surgery and their rigidity progressively decreases over time as the fusion process progresses. Resorption of the rods decreases the stress at adjacent segments without the need for further surgery. Further studies will consider multi-level fusion models implanted with biodegradable rods.
